# Selection of Essential Neural Activity Timesteps for Intracortical Brain–Computer Interface Based on Recurrent Neural Network

**DOI:** 10.3390/s21196372

**Published:** 2021-09-24

**Authors:** Shih-Hung Yang, Jyun-We Huang, Chun-Jui Huang, Po-Hsiung Chiu, Hsin-Yi Lai, You-Yin Chen

**Affiliations:** 1 Department of Mechanical Engineering, National Cheng Kung University, Tainan City 701, Taiwan; z10806026@ncku.edu.tw (J.-W.H.); n16091485@gs.ncku.edu.tw (C.-J.H.); n16090065@gs.ncku.edu.tw (P.-H.C.); 2 Key Laboratory of Medical Neurobiology of Zhejiang Province, Department of Neurology of the Second Affiliated Hospital, Interdisciplinary Institute of Neuroscience and Technology, Zhejiang University School of Medicine, Zhejiang University, Hangzhou 310027, China; laihy@zju.edu.cn; 3 Key Laboratory for Biomedical Engineering of Ministry of Education, College of Biomedical Engineering and Instrument Science, Zhejiang University, Hangzhou 310027, China; 4 Department of Biomedical Engineering, National Yang Ming Chiao Tung University, Taipei 112, Taiwan; irradiance@so-net.net.tw

**Keywords:** intracortical brain–computer interface, recurrent neural network, temporal attention module, timestep selection

## Abstract

Intracortical brain–computer interfaces (iBCIs) translate neural activity into control commands, thereby allowing paralyzed persons to control devices via their brain signals. Recurrent neural networks (RNNs) are widely used as neural decoders because they can learn neural response dynamics from continuous neural activity. Nevertheless, excessively long or short input neural activity for an RNN may decrease its decoding performance. Based on the temporal attention module exploiting relations in features over time, we propose a temporal attention-aware timestep selection (TTS) method that improves the interpretability of the salience of each timestep in an input neural activity. Furthermore, TTS determines the appropriate input neural activity length for accurate neural decoding. Experimental results show that the proposed TTS efficiently selects 28 essential timesteps for RNN-based neural decoders, outperforming state-of-the-art neural decoders on two nonhuman primate datasets ($R^{2}=0.76\pm 0.05$ for monkey Indy and CC=0.91±0.01 for monkey N). In addition, it reduces the computation time for offline training (reducing 5–12%) and online prediction (reducing 16–18%). When visualizing the attention mechanism in TTS, the preparatory neural activity is consecutively highlighted during arm movement, and the most recent neural activity is highlighted during the resting state in nonhuman primates. Selecting only a few essential timesteps for an RNN-based neural decoder provides sufficient decoding performance and requires only a short computation time.

## 1. Introduction

Intracortical brain–computer interfaces (iBCIs) aim to improve the daily lives of paralyzed patients by restoring their motor functions [1,2]. An iBCI ascertains the patient’s movement intention and generates motor commands for assistive devices, such as computer cursors [3], and the functional electrical stimulation of paralyzed limbs [4]. The iBCI first records neural activity by microelectrode arrays implanted in motor-related brain regions, such as the primary motor cortex. Then, a neural decoder translates neural activity into movement intention or spatial location information. Conventional neural decoding techniques process well-segmented neural activities in previous time windows where task-related information is likely encoded as spiking sequences. Thus, neural decoding may benefit from continuous neural activity because movement intention often occurs before execution [5]. Therefore, a sequence of spike count vectors from many preceding time windows is usually adopted to capture the neural response dynamics [6] and improve the decoding accuracy [7]. However, an excessively long neural sequence may be polluted by stochastic noise, and a short neural sequence may not contain sufficient information. Furthermore, the length of the neural sequence for a neural decoder presents inter-subject variability and often depends on the task. Thus, the length of the neural sequence, a hyperparameter for decoding, is usually designed manually by trial and error. In the literature, various terminologies are used for the length of a neural sequence, including number of timesteps [8,9], number of taps [6,10], length of the sliding window [4] and time window size [7,11]. We refer to the length of a neural sequence for decoding as the number of timesteps for consistency with most neural decoders and considering its use in neuroscience [12].

Recent advances in recurrent neural networks (RNNs) have led to improved neural decoder designs [9,13] and real-time BCI systems [14]. RNN-based neural decoders learn the neural response dynamics from the neural activity in both the previous and current timesteps (time bins) [6]. These decoders also employ gates to balance the contributions of the current and previous timesteps when learning a neural sequence. Although most RNN hyperparameters have been analyzed and discussed in a large-scale study [15], obtaining the correct number of timesteps for neural decoding remains challenging.

Existing iBCIs often rely on grid search to optimize the number of timesteps. Alternatively, a high number of timesteps can be selected to retain informative timesteps. Table 1 summarizes the various iBCIs that use RNNs as the neural decoders for nonhuman primate models. In most of the studies, the long short-term memory (LSTM) architecture is applied as an RNN for neural decoding. These iBCIs predict the kinematic states based on the neural activity over the previous 2–30 timesteps.

**Figure 1:**
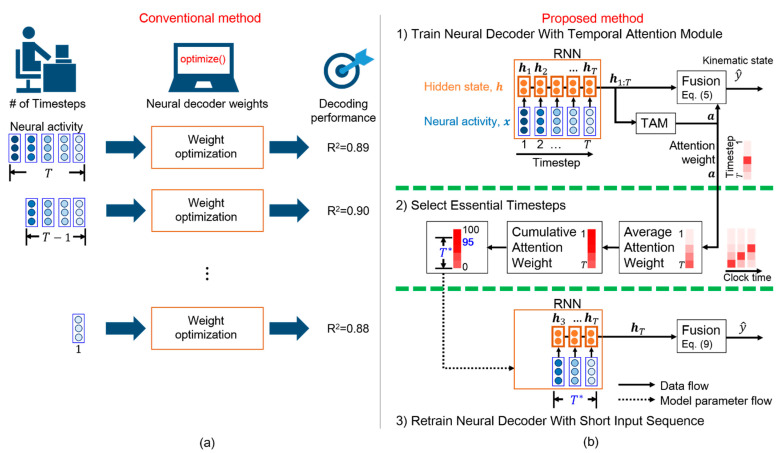
Selection of essential timesteps for neural decoding. (**a**) Conventional method consisting of numerous iterations with high computational cost (*R*^2^, coefficient of determination). (**b**) Proposed temporal attention-aware timestep selection (TTS) method. This one-pass method only requires three steps. First, the neural decoder is trained with the temporal attention module (TAM). Next, essential timesteps ($T^{*}$) are selected according to the attention weights. Finally, the neural decoder with a short input sequence of length $T^{*}$ is retrained.

As the number of timesteps varies according to the task and subject, grid search is commonly used to determine the appropriate number of timesteps. Grid search evaluates all the possible numbers of timesteps and selects the number that provides the best fit, as shown in Figure 1a. Grid search evaluates the decoding performance according to the number of timesteps. One possible number of timesteps is designated to a neural decoder, and the model weights of the neural decoder are then optimized. Subsequently, a score is determined to evaluate the effectiveness of the neural decoder for the selected number of timesteps. However, grid search may cause a high computational burden because weight optimization is computationally expensive over all the parameter combinations. Consequently, grid search has a low applicability in real-world iBCIs.

Alternatively, a high number of timesteps can be used in a neural decoder [6], assuming that contextual information related to movement intention is likely available over such a long period. To efficiently extract task-related features, RNNs can decode neural activity over several timesteps [6]. However, excessively long neural activity periods cause a high computational burden because an RNN iteratively updates the hidden states at each timestep. The iterative processing in the RNN cannot be implemented in parallel, which increases the computational burden. Moreover, long neural activity periods may introduce stochastic noise, which hinders the decoding performance. Therefore, an adequate number of timesteps can reduce the computational burden and enable real-time iBCIs. However, the lack of meaningful interpretation for each timestep impedes the selection of an adequate number of input timesteps for decoding.

Ensuring that timesteps are interpretable by a neural decoder is essential for selecting an adequate number of input timesteps. In other words, one can easily select essential timesteps according to the interpretable salience of each timestep in a neural sequence. Because the RNN models the temporal dynamics of neural sequences, this architecture is widely used as the basis for neural decoders. In fact, RNNs employ gates to individually process each timestep and balance the contribution of the neural activity in the current and previous timesteps. Nevertheless, the relative importance of the timesteps is difficult to interpret [18]. To improve the interpretability of models, attention modules can be included to highlight salient information [19]. Attention modules have been demonstrated to enhance salient objects in images [20] and localize actions in videos [21]. In particular, a temporal attention module (TAM) highlights the time intervals of event occurrences by exploiting the relations in features over time [22]. The TAM provides a sequence of attention weights in which each element corresponds to a timestep and whose values represent the relevance of the timestep to an event of interest. Furthermore, the TAM highlights salient timesteps relevant to the final decision and ignores noisy timesteps in an input sequence. Therefore, we include the TAM in the RNN-based neural decoder to highlight salient timesteps that contribute to determining the kinematic states for increasing interpretability.

Inspired by the TAM that highlights the time intervals of interest, we propose a temporal attention-aware timestep selection (TTS) method that reduces the design complexity and computational burden in an RNN-based neural decoder. To the best of our knowledge, this is the first method that automatically selects essential timesteps for RNN-based neural decoding. We adopt the TAM to determine the contribution of neural activity at each timestep to the prediction of movement intention, assuming that this contribution varies across timesteps. Determining the relative importance of each timestep in a behavioral task can improve the interpretability of timesteps and facilitate timestep selection for neural decoding.

The main contributions of this study are summarized as follows:

1. We propose a scheme that efficiently determines the essential timesteps for a general RNN-based neural decoder, thus avoiding the time-consuming grid search;
2. We adopt a TAM that interprets the salience of each timestep for predicting movement intention, leading to essential timestep selection;
3. Experimental results reveal that the proposed TTS can determine the essential timesteps for three RNN-based neural decoders while reducing the computation time of offline training and online prediction;
4. The RNN-based neural decoders with a few essential timesteps outperform state-of-the-art neural decoders on two nonhuman primate datasets;
5. The visualization of attention weights demonstrates that only a few neural activity timesteps are emphasized for neural decoding during arm motion and resting.

## 2. Behavioral Tasks and Neural Data Collection

In this study, we used publicly available nonhuman primate datasets acquired from monkey Indy [23] and monkey N [24].

### 2.1. Monkey Indy

**Figure 2:**
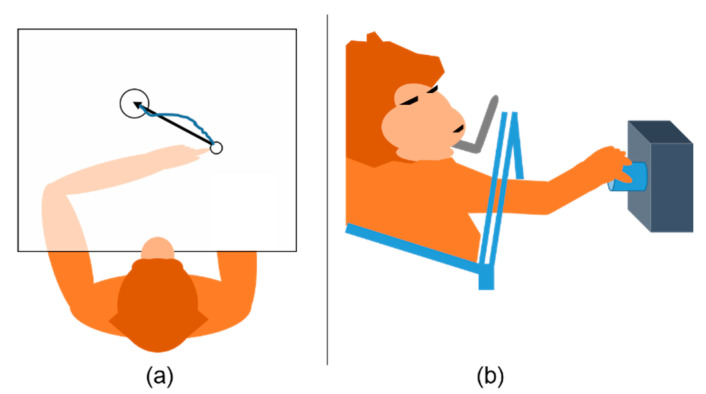
Behavioral tasks. (**a**) Monkey Indy reached a target presented on a horizontal plane. An opaque barrier directly blocked visual feedback from the arm so that monkey Indy could not see his arm. The black arrow presents the movement vector when monkey Indy started to reach the target. The blue curve shows the motion trajectory. (**b**) Monkey N performed an instructed delayed reach-to-grasp task by grasping an object with a side grip and then pulling it.

Monkey Indy (*Macaca mulatta*) was trained to reach targets randomly appearing on a plane using its left arm, as illustrated in Figure 2a. Monkey Indy successfully reached the target when placing the fingertip on the target for 450 ms. Then, a new target was randomly drawn on any possible locations. The fingertip position was acquired by an electromagnetic position sensor, and neural activity was recorded using a 96-channel silicon microelectrode array (Blackrock Microsystems, Salt Lake City, UT, USA), which was chronically implanted in the primary motor cortex (M1). This array was designed to cover the upper-arm and shoulder representation areas. The neuronal activity was sorted using a custom software written in C++, where 171–413 units were obtained. The population firing rate was computed at a bin width of 64 ms and was considered to be the input of a neural decoder. The population firing rate, $x\in {\mathbb{R}}^{C}$, had *C* elements, with *C* being either the number of channels or single units (single neurons). Data from three to seven sessions over 300 days were collected. More details about the corresponding behavioral tasks and neural data collection can be found in [23,25].

### 2.2. Monkey N

Monkey N (*Macaca mulatta*) was trained to perform an instructed delayed reach-to-grasp task, as illustrated in Figure 2b. Monkey N grasped an object using either a side grip or a precision grip and then pulled the object against one of two possible loads by applying either a high or low force. The horizontal displacement of the object was measured using a Hall effect sensor. A 96-channel Utah electrode array (Blackrock Microsystems, Salt Lake City, UT, USA) was chronically implanted to cover the primary motor cortex (M1) and dorsal or ventral premotor cortex. The neuronal activity was sorted using the Plexon Offline Sorter (Plexon, Dallas, TX, USA), where 156 units were obtained. As the trials contained large portions of preparatory delay intervals, they were segmented to contain only the motion execution intervals. Nevertheless, only the data from one session are publicly available. More details about the corresponding behavioral tasks and neural data collection can be found in [24].

## 3. Temporal Attention-Aware Timestep Selection for RNN-Based Neural Decoder

We aimed to efficiently determine the essential timesteps for an RNN-based neural decoder. Figure 1b illustrates the proposed TTS. Most kinematic information is assumed to be encoded over a few essential timesteps in a long neural sequence. First, a long neural sequence is fed into the neural decoder with a TAM, which learns the attention weights that provide the meaningful salience of each timestep. The salient timesteps are likely related to movement intention. Then, a statistical analysis enables the determination of the number of timesteps required for the neural decoder. Next, the neural decoder is modified by the selected timesteps and retrained for a few epochs. Finally, a neural decoder with the adequate number of input timesteps is obtained. We share our code and models at https://github.com/nclab-me-ncku/Temporal_Attention_LSTM (accessed on 1 September 2020) [26].

### 3.1. RNN-Based Neural Decoder

For an RNN-based neural decoder, let the input sequence $x=\left\{x_{t-T+1},\cdots ,x_{t}\right\}$ contain the *t*-th observation of neural activity and the previous *T* timesteps of neural activity. For ease of notation and without loss of generality, we refer to this input sequence as $x=\left\{x_{1},\cdots ,x_{T}\right\}$, where $x_{\tau }\in {\mathbb{R}}^{C}$ represents neural activity with *C* elements (population firing rate) at the τ-th timestep.

An RNN is adopted to obtain a hidden representation embedded in the observed neural activity. The hidden representation consists of sequence $h_{1},\cdots ,h_{T}$ of length *T*, where $h_{\tau }\in {\mathbb{R}}^{M}$ denotes a hidden state at the τ-th timestep. Once a bidirectional RNN is implemented, the hidden state consists of both forward hidden state ${\vec{h}}_{\tau }\in {\mathbb{R}}^{M}$ and backward hidden state ${\overset{\leftarrow }{h}}_{\tau }\in {\mathbb{R}}^{M}$ (i.e., $h_{\tau }=\left[{\vec{h}}_{\tau },{\overset{\leftarrow }{h}}_{\tau }\right]\in {\mathbb{R}}^{2M}$). Mathematically, the hidden state can be computed as follows:

(1) $$h_{\tau }=f\left(x_{\tau },h_{\tau -1}\right),$$

where f(·) represents the RNN model. The RNN can be the vanilla RNN [27], LSTM [28], or gated recurrent unit (GRU) [29], which have been widely adopted for neural decoding [4,6,7].

### 3.2. Temporal Attention Module

The TAM aims to determine the relative importance of each neural activity timestep. The relative importance is then used to select the essential timesteps for neural decoding. To model the relative importance, attention weight $a_{\tau }$ is estimated as follows:

(2) $$u_{\tau }=\mathrm{ReLU}\left(Wh_{\tau }+b\right),$$

(3) $$a_{\tau }=\frac{\exp(u_{\tau }^{T}v)}{\sum_{\tau =1}^{T}\exp(u_{\tau }^{T}v)},$$

where W, b, v, and ReLU denote a linear transformation matrix, bias term, linear transformation vector, and rectified linear unit, respectively. Equation (3) represents a softmax function that normalizes attention weights in [0,1].

The TAM aggregates the hidden states of all timesteps according to the attention weights to form the following hidden representation:

(4) $$h_{w}=\sum_{\tau =1}^{T}a_{\tau }h_{\tau }.$$

This learned hidden representation is then fed into a fully connected layer to predict a kinematic state as follows:

(5) $$\hat{y}=d^{T}\cdot h_{w},$$

where d denotes a linear transformation vector and $\hat{y}$ can represent position, velocity, or acceleration.

### 3.3. Essential Timestep Selection for Neural Decoding

**Figure 3:**
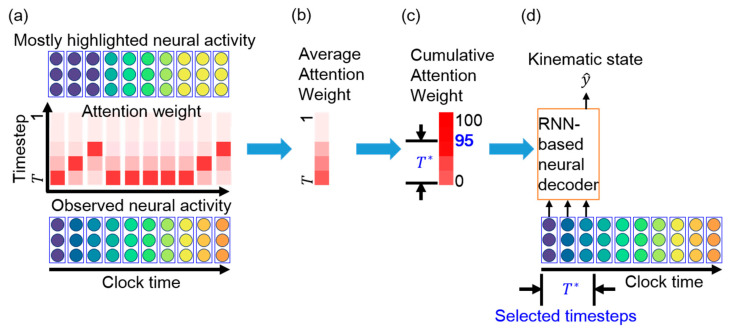
Schematic of essential timestep selection using attention weights. (**a**) Observed neural activity (bottom) and attention weights (middle) across clock time. The mostly highlighted neural activity at each time bin (top) is obtained from the attention weight. For example, the mostly highlighted neural activity at the first time bin is the neural activity at timestep T, which is observed at the first time bin. The mostly highlighted neural activity at the second time bin is the neural activity at timestep T−1, which is identical to the neural activity observed at the first time bin. Therefore, the mostly highlighted neural activities at the first three time bins are identical to the observed neural activity at the first time bin. (**b**) Average attention weight across clock time. (**c**) Cumulative attention weight over the timestep. The most recent $T^{*}$ consecutive timesteps are essential for neural decoding. (**d**) The RNN-based neural decoder is retrained with $T^{*}$ timesteps.

Our aim is to select only essential consecutive timesteps for the RNN-based neural decoder. As the attention weights reflect the relevance of the neural activity information in the corresponding timesteps, the cumulative attention weights determine the importance of the consecutive timesteps for neural decoding. For a high cumulative value, the corresponding consecutive timesteps are suitable for the RNN-based neural decoder. In this case, the neural decoder can accurately predict the kinematic state from the neural activity observed over these few consecutive timesteps while reducing the computation time. Subsequently, the most recent $T^{*}$ consecutive timesteps are selected for neural decoding, where $T^{*}\leq T.$ Figure 3 schematically illustrates the essential timestep selection process using attention weights.

The value of $T^{*}$ is determined in three steps by using the cumulative percentage of the attention weights. First, as the neural decoder predicts kinematic state ${\hat{y}}_{t}$ based on the *t*-th observation, the corresponding attention weights are $a^{t}={\left[a_{1}^{t},\;a_{2}^{t},\ldots ,a_{T}^{t}\right]}^{T}.$ Second, the average attention weight at timestep τ over all the observations (i.e., over clock time) is determined as follows:

(6) $${\bar{a}}_{\tau }=\frac{1}{N}\sum_{t=1}^{N}a_{\tau }^{t},$$

where *N* is the number of observations. Then, the average attention weights are obtained as

(7) $$\bar{a}={\left[{\bar{a}}_{1},{\bar{a}}_{2},\ldots ,{\bar{a}}_{T}\right]}^{T},$$

Third, $T^{*}$ is selected as the minimum that makes

(8) $$a^{*}=100\%\cdot \sum_{\tau =T-T^{*}+1}^{T}{\bar{a}}_{\tau }/\sum_{\tau =1}^{T}{\bar{a}}_{\tau },$$

to be above a predefined threshold. We select this threshold to be 95% based on principal component analysis [30], where the first few principal components represent over 95% of the total variation in a signal. Accordingly, we aim to retain the timesteps that represent 95% of the attention weights. In these timesteps, the corresponding neural activity likely encodes the most relevant information about a kinematic state.

### 3.4. Neural Decoder Retraining with Short Input Sequence

Once $T^{*}$ is obtained, the input sequence is modified to $x^{*}=\left\{x_{T-T^{*}+1},\cdots ,x_{T-1},x_{T}\right\}$, which has a shorter length $T^{*}$ ($T^{*}<T$). The RNN-based neural decoder is then retrained using the modified input sequence. Note that the TAM is no longer used in the retrained neural decoder. The kinematic state is predicted as follows:

(9) $$\hat{y}=d^{T}\cdot h_{T},$$

where $h_{T}$ denotes the hidden state at timestep *T*. Whereas (5) aggregates the hidden states only at all timesteps to predict the kinematic state, while (9) only aggregates the hidden state at the final timestep. We observed no difference between the two methods in terms of decoding performance. However, most existing RNN-based neural decoders adopt (9) because its computational complexity is lower than that of (5). Similarly, we adopt (9) for the neural decoding with short input sequences.

### 3.5. Optimization

The neural decoder aims to predict a kinematic state from neural activity. We use the squared L2 norm (mean squared error) as the loss function:

(10) $$L=\sum_{y\in B}{\left(y-\hat{y}\right)}^{2},$$

where y and $\hat{y}$ denote the actual and predicted kinematic states, respectively, and B denotes the mini-batch. To optimize the model weights, we adopt Adam [31] with a learning rate of $10^{-4}$. The coefficients used for computing the running averages of the gradient and their squares are 0.9 and 0.999, respectively, to achieve a high generalization ability to various RNNs.

### 3.6. Performance Evaluation and Statistical Evaluation

We evaluated the decoding performance using the coefficient of determination ($R^{2}$) [25] and Pearson’s correlation coefficient (CC) [8]. The coefficient of determination measures the goodness of fit of a neural decoder as follows:

(11) $$R^{2}=1-\frac{\mathrm{Var}\left(y-\hat{y}\right)}{\mathrm{Var}\left(y\right)},$$

where Var(·) is the variance. The Pearson’s correlation coefficient measures the linear correlation between the actual and predicted kinematic states as follows:

(12) $$CC=\frac{\sigma_{y\hat{y}}}{\sigma_{y}\sigma_{\hat{y}}},$$

where $\sigma_{y\hat{y}}$ represents the covariance between y and $\hat{y}$, and $\sigma_{y}$ and $\sigma_{\hat{y}}$ represent the standard deviations of y and $\hat{y}$, respectively. For monkey Indy, the proposed TTS was compared with the state-of-the-art method in [25] that uses the coefficient of determination. For monkey N, the proposed TTS was compared with state-of-the-art method in [8] that uses Pearson’s correlation coefficient.

For rigorous statistical evaluation, the Shapiro Wilk test was first performed to assess whether the observations generated from a normal distribution because it is suggested to be the most powerful normality test [32]. The Friedman test is a non-parametric equivalent of the repeated-measures analysis of variance (ANOVA) and does not assume that the observations are normally distributed. Therefore, the Friedman ANOVA test, followed by a *post hoc* Wilcoxon signed-rank test, was applied to assess the effect of various lengths *T* in the timestep selection. The significance levels were set at * *p* < 0.05, ** *p* < 0.01, and *** *p* < 0.001. This statistical evaluation has been widely adopted in the neuroscience community [33]. Furthermore, the Friedman ANOVA test, followed by a *post hoc* Wilcoxon signed-rank test, was applied to assess the decoding performance compared with state-of-the-art methods.

## 4. Experimental Results

### 4.1. Implementation Details

We implemented three RNN-based neural decoders that are widely used in existing iBCIs: vanilla RNN, LSTM, and GRU. Vanilla RNN predicts the output according to both the input sequence and hidden states. However, it suffers from the vanishing gradient problem during training. LSTM avoids this problem by introducing a forget gate that controls the influence of short- and long-term dependencies. GRU further simplifies the gates to reduce the computational cost. Table 2 lists the settings for the three neural decoders. The neural decoders predicted the velocity of the hand movement for both monkey Indy and monkey N in this study. As training involved random weight initialization, each architecture was executed in five independent runs. The experiments were implemented using the PyTorch library and executed on a computer equipped with an NVIDIA GeForce GTX 1080 Ti GPU.

The training and testing protocols for the monkey Indy and monkey N data were the same as those used in [25] and [8], respectively. For monkey Indy, the first 5000 samples of each session were used to establish the training set, and the remaining samples were used as the test set. The neural decoder was independently trained during each session. For monkey N, the data, excluding large portions of preparatory delay intervals, were split into ten folds, where eight folds (1178 samples), one fold (147 samples), and one fold (148 samples) established the training, validation, and test sets, respectively.

### 4.2. Timestep Selection for Neural Decoders

Given an input neural sequence of length *T*, the TTS highlighted the neural activities and selected the essential timesteps $T^{*}$ for the RNN-based neural decoder. We quantitatively evaluated the proposed TTS for three RNN-based neural decoders and the effects of various lengths *T*.

**Figure 4:**
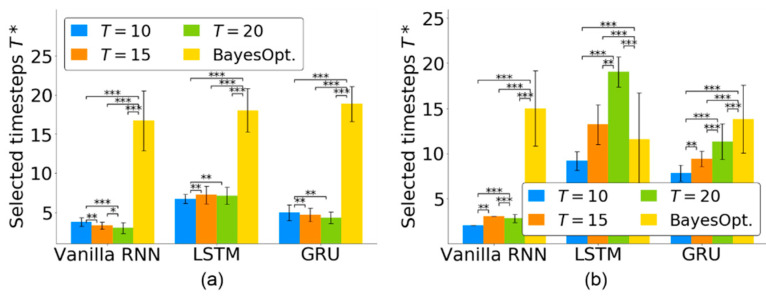
Number of selected timesteps $T^{*}$ in three RNN-based neural decoders for neural activity of length T=10, 15, 20 from (**a**) monkey Indy and (**b**) monkey N. Each bar represents the mean, and the whiskers represent the standard deviation of $T^{*}$ across 37 sessions over 300 days for monkey Indy and across five independent runs for monkey N. $T^{*}$ was independently obtained in each session. BayesOpt. represents the Bayesian optimization. * *p* < 0.05, ** *p* < 0.01, and *** *p* < 0.001.

For the quantitative evaluation of the timestep selection, Figure 4 shows the selected timesteps $T^{*}$ of the three RNN-based neural decoders for T=10, 15, 20 using the monkey Indy data in [23] and the monkey N data in [24]. For monkey Indy, Figure 4a shows that each RNN-based neural decoder selected fewer than seven timesteps. For the vanilla RNN, significantly fewer timesteps ($T^{*}=3.00\pm 0.71$) was selected from T=20 than that from T=10 and 15 (analyzed by Friedman ANOVA test, followed by Wilcoxon signed-rank test). For the LSTM, significantly fewer timesteps ($T^{*}=6.70\pm 0.57$) was selected from T=10 than that from T=15 and 20 (analyzed by Friedman ANOVA test, followed by Wilcoxon signed-rank test). For the GRU, significantly fewer timesteps ($T^{*}=4.30\pm 0.76$) was selected from T=20 than that from T=10 and 15 (analyzed by Friedman ANOVA test, followed by the Wilcoxon signed-rank test). There was a significant difference among the groups T=10, 15, 20 for the vanilla RNN (*p* < 0.001, Friedman ANOVA test), LSTM (*p* < 0.001, Friedman ANOVA test) and GRU (*p* < 0.001, Friedman ANOVA test).

For monkey N, Figure 4b shows that the proposed TTS selected fewer than three timesteps for the vanilla RNN. For the vanilla RNN, significantly fewer timesteps ($T^{*}=2.00\pm 0$) was selected from T=10 than that from T=15 and 20 (analyzed by Friedman ANOVA test, followed by Wilcoxon signed-rank test). For the LSTM, significantly fewer timesteps ($T^{*}=9.17\pm 1.04$) was selected from T=10 than that from T=15 and 20 (analyzed by Friedman ANOVA test, followed by Wilcoxon signed-rank test). For the GRU, significantly fewer timesteps ($T^{*}=7.96\pm 0.73$) was selected from T=10 than that from T=15 and 20 (analyzed by Friedman ANOVA test, followed by Wilcoxon signed-rank test). There was a significant difference among the groups T=10, 15, 20 for the vanilla RNN (*p* < 0.001, Friedman ANOVA test), LSTM (*p* < 0.001, Friedman ANOVA test) and GRU (*p* < 0.001, Friedman ANOVA test).

Our proposed TTS was compared to the Bayesian optimization [34], which is widely used to search effective timesteps for neural decoding. Figure 4 shows that our TTS could select significantly fewer timesteps for vanilla RNN and GRU than that achieved by the Bayesian optimization (*p* < 0.001 for T=10, 15, 20, Friedman ANOVA test, followed by the Wilcoxon signed-rank test). The Bayesian optimization selected fewer timesteps than that achieved by the LSTM (*p* < 0.001 for T=15, 20, Friedman ANOVA test, followed by the Wilcoxon signed-rank test). However, the LSTM optimized by the Bayesian optimization achieved a lower decoding performance than that optimized by the TTS (see Section 4.4). The TTS was effective in selecting essential timesteps for neural decoders.

### 4.3. Timestep Selection across Multiple Sessions

In Section 4.2, we quantitatively evaluated the effectiveness of TTS for monkey Indy. As the neural activity of monkey Indy was collected across 37 sessions over 300 days, we also evaluated the variation of $T^{*}$ over the sessions. This evaluation could not be conducted for monkey N because only one of its sessions is publicly available.

**Figure 5:**
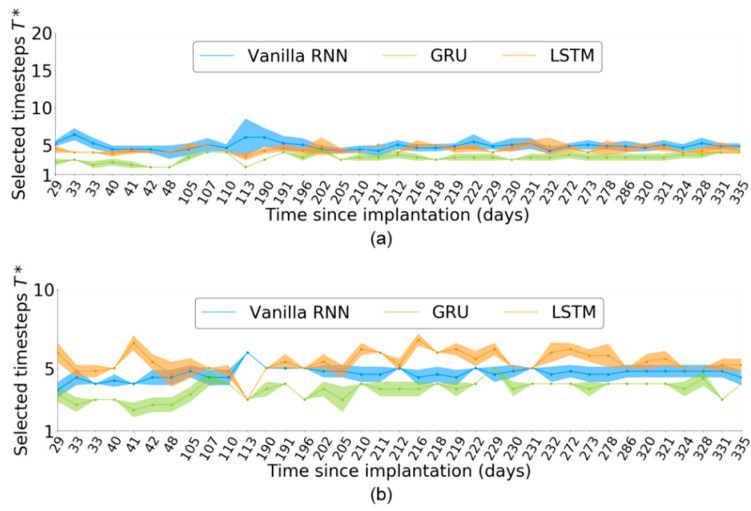
Number of selected timesteps, $T^{*}$, across 37 sessions corresponding to neural activity of length (**a**) T=10 and (**b**) T=20. The shaded areas represent the $T^{*}$ distributions obtained from five independent runs per session.

Figure 5 shows $T^{*}$ across the 37 sessions for each RNN-based neural decoder. $T^{*}$ slightly varied across the sessions. Given an input neural sequence of length T=10, the timestep selection for the vanilla RNN was more stable than that for LSTM and GRU across all the 37 sessions. Even within a single session, $T^{*}$ slightly varied across independent runs, as indicated by the $T^{*}$ distributions defined by the shaded areas of Figure 5. This finding suggests that $T^{*}$ may be affected by the random initial weights of the neural decoder in each independent run.

For T=20, the variation of $T^{*}$ was larger than that for T=10 in the RNN-based neural decoders. Furthermore, both the GRU and LSTM obtained a more stable $T^{*}$ than the vanilla RNN, which differed from the case of T=10.

### 4.4. Comparison with State-of-the-Art Methods

**Figure 6:**
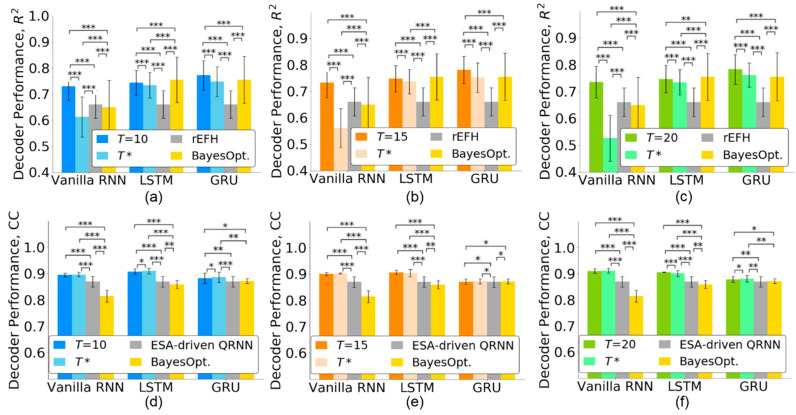
Decoding performance of the state-of-the-art methods for the monkey Indy (top row) and monkey N (bottom row) data. The neural decoders were first trained with T timesteps and then retrained with $T^{*}$ timesteps. (**a**,**d**) T=10. (**b**,**e**) T=15. (**c**,**f**) T=20. The error bars indicate standard deviations across 37 sessions for the monkey Indy data and across five independent runs for the monkey N data. BayesOpt. represents the Bayesian optimization. The significance levels were set at * *p* < 0.05, ** *p* < 0.01, and *** *p* < 0.001.

When the number of timesteps is reduced to $T^{*}$, the decoding performance may be affected. Thus, we evaluated the effect of timestep selection for three RNN-based neural decoders. Furthermore, we compared the decoding performance with state-of-the-art methods including recurrent exponential-family harmonium (rEFH) [25] for the monkey Indy data and the entire spiking activity-driven quasi-RNN (ESA-driven QRNN) [8] for the monkey N. Figure 6a–c show that when the number of timesteps was reduced from T to $T^{*}$, all the neural decoders reduced their performance for monkey Indy. However, the decoding performances of both the LSTM and GRU ($R^{2}=0.74\pm 0.05$ for LSTM and $R^{2}=0.76\pm 0.05$ for GRU) were significantly better than that achieved by the rEFH in [25] (*p* < 0.001 for T=10, 15, 20, Friedman ANOVA test, followed by the Wilcoxon signed-rank test). The vanilla RNN with $T^{*}$ timesteps achieved a lower decoding performance than the rEFH.

For the monkey N, the decoding performance of the three RNN-based neural decoders did not decrease when the number of timesteps was reduced to $T^{*}$, as shown in Figure 6d–f. Both vanilla RNN and LSTM with $T^{*}$ timesteps (CC=0.91±0.01 for vanilla RNN and CC=0.91±0.01 for LSTM) significantly outperformed the ESA-driven QRNN [8] (*p* < 0.001 for T=10, 15, 20, Friedman ANOVA test, followed by the Wilcoxon signed-rank test). The GRU achieved significantly better decoding performance (CC=0.89±0.02) than the ESA-driven QRNN (*p* < 0.001 for T=10, *p* < 0.05 for T=15, and *p* < 0.01 for T=20, Friedman ANOVA test, followed by the Wilcoxon signed-rank test).

We further compared the decoding performance of the neural decoders whose timesteps were optimized by the Bayesian optimization [34] and the TTS. Figure 6 shows that both vanilla RNN and LSTM optimized by the Bayesian optimization achieved lower decoding performance than those optimized by the TTS (*p* < 0.001 for T=10, 15, 20, Friedman ANOVA test, followed by the Wilcoxon signed-rank test). The GRU, optimized by the Bayesian optimization, achieved a decoding performance comparable with that achieved by the TTS. However, the Bayesian optimization selected longer timesteps than the TTS for the GRU, as shown in Figure 4. The TTS could not only select fewer timesteps but also facilitate a higher decoding performance than the Bayesian optimization.

### 4.5. Reduced Computation Time

We also evaluated the computational efficiency of the proposed TTS by obtaining the computation time as listed in Table 3. For fair comparison, the computation time needed to train a neural decoder for one epoch during the training phase and that needed to predict one kinematic state during the testing phase are reported. Table 3 shows that the proposed TTS reduced the computation time for all the RNN-based neural decoders. The vanilla RNN and LSTM could reduce the computation time by at least 16% for online prediction, possibly enabling real-time iBCI operation. Furthermore, the computation time could be reduced by 5–12% for offline training. The time duration of the timesteps selection achieved by our TTS was compared to the Bayesian optimization [34], which is a conventional optimization approach. The rightmost two columns of Table 3 show that the TTS could save 48–81% of the time duration of the timesteps selection compared with the Bayesian optimization. This demonstrates the computational efficiency of our TTS method.

### 4.6. Visualization of Attention Weights in TTS

**Figure 7:**
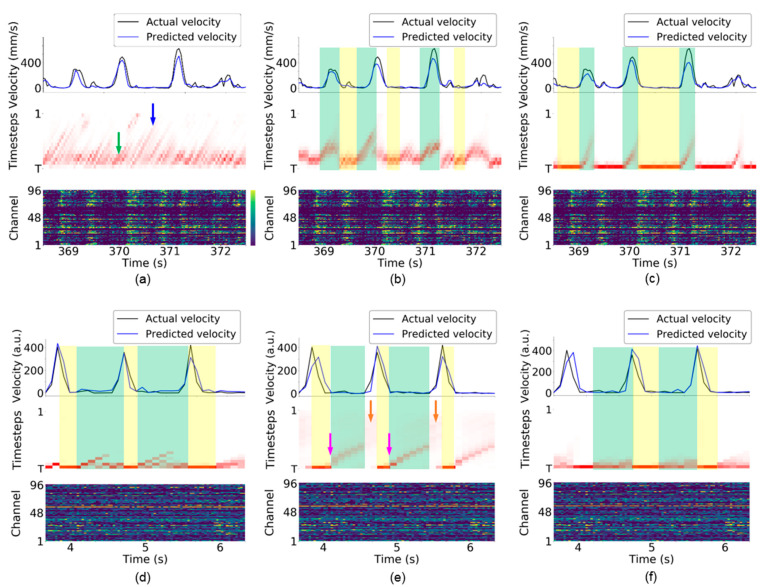
Visualization of attention weights for monkey Indy [23] (top row) and monkey N [24] (bottom row). (**a**,**d**) Vanilla RNN. (**b**,**e**) LSTM. (**c**,**f**) GRU. Each subfigure presents the kinematic state (top), attention weights (middle), and neural activity (bottom). The top panel of each subfigure presents the Euclidean norm of the velocity vector ${\left[v_{x},v_{y}\right]}^{T}$. In the bottom panel of each subfigure, the firing rate (neural activity) was recorded from a 96-electrode array. For the middle panel of each subfigure, the vertical axis indicates the timesteps used in the RNN-based neural decoder, and the horizontal axis indicates the clock time. The green and blue arrows indicate that the neural decoder highlights shorter and longer timesteps, respectively, in (**a**). The pink arrow indicates the early preparatory period in (**e**). The orange arrow indicates that the attention weights at all timesteps have similar color in (**e**), suggesting that these attention weights have similar values. The green-shaded area indicates that the neural decoder consecutively highlights the neural activity observed in the previous timesteps. The yellow-shaded area indicates that the neural decoder highlights the recently observed neural activity. The examples were obtained from session indy_20161024_03 for monkey Indy and session i140703-001 for monkey N.

The attention weights of the TAM in TTS were visualized across all observations, as shown in Figure 7. Three RNN-based neural decoders were implemented using neural activity over 15 timesteps (i.e., T=15) for the monkey Indy data [23] and monkey N data [24]. Different attention weights were obtained from the vanilla RNN, LSTM, and GRU. Furthermore, the attention weights also differed between behavioral tasks.

The attention weights implicitly reveal many line segments with a positive slope and various lengths. Figure 3a illustrates the meaning of the line segments. At the first time bin, the TAM highlights the neural activity at timestep T, which is the most recently observed neural activity. At the second time bin, the TAM highlights the neural activity at timestep T−1, which represents identical neural activity to that highlighted at the first time bin. The top panel of Figure 3a shows that the TAM highlights identical neural activity to that observed in the first time bin (the leftmost part in purple in the bottom panel) across the first three time bins. The attention weights with large values could be fitted with a straight-line segment. This suggests that, although new neural activity is observed, the neural decoder prefers to decode the leftmost neural activity rather than the most recently observed neural activity. A longer line segment indicates that neural activity within a time window is successively highlighted over a longer period. Thus, the highlighted neural activity is likely important over this period for neural decoding. This further implies that most highlighted timesteps are potentially related to the kinematic state and are thus selected as essential timesteps for neural decoding.

For the vanilla RNN-based neural decoder, multiple timesteps are highlighted at each time bin, as shown in Figure 7a (i.e., numerous attention weights are large in each column in the middle panel) for monkey Indy. Hence, the vanilla RNN relies on multiple previous neural activity data for neural decoding. The vanilla RNN dynamically requires shorter and longer timesteps for neural decoding, as indicated by the green and blue arrows in Figure 7a, respectively. For monkey N, the preparatory neural activity observed at the leftmost time bin in the green-shaded area in Figure 7d is consecutively highlighted during the preparatory period and arm acceleration period where monkey N was instructed to grasp and pull an object. In addition to the preparatory neural activity, the recent two timesteps are highlighted simultaneously; however, the most recent timestep is highlighted during the arm deceleration period, as indicated by the yellow-shaded areas where monkey N was allowed to release the object.

In the LSTM-based neural decoder, attention weights have more regular patterns compared with those of the vanilla RNN-based neural decoder for monkey Indy. Figure 7b shows that approximately five timesteps are highlighted at each time bin. When monkey Indy performed the reaching movement, the attention weights implicitly revealed thick line segments, as indicated by the green-shaded areas. According to the illustration of the line segments in Figure 3a, the thick line segment suggests that the neural activity observed at multiple time bins are consecutively highlighted and are important for neural decoding over this reaching period. In other words, the preparatory neural activity that occurred before the movement onset is consecutively highlighted during the arm movement. In contrast, the neural activity observed at the previous one timestep was highlighted during the resting state, as indicated by the yellow-shaded areas in Figure 7b.

For monkey N, the neural activity observed during the early preparatory period (as indicated by the pink arrows) was consecutively highlighted during the preparatory period, as indicated by the green-shaded areas in Figure 7e. The attention weights implicitly revealed a thinner line segment than that for monkey Indy. This suggests that neural decoding of the instructed delayed reach-to-grasp task highlights fewer timesteps compared with the neural decoding of the reaching task, as can be seen from Figure 7b. The values of the attention weights during the arm acceleration period are small (i.e., the attention weights at all timesteps are almost white), as indicated by the orange arrows. This suggests that the LSTM-based neural decoder treats the neural activity at all timesteps equally. This makes it difficult for TTS to select fewer essential timesteps for neural decoding, which leads to a high number of selected timesteps, as shown in Figure 4b. During the arm deceleration period, the neural activity observed at the most recent timestep is highlighted, as indicated by the yellow-shaded areas in Figure 7b.

For the GRU-based neural decoder, the most recently observed neural activity was highlighted during the resting state for monkey Indy, as indicated by the yellow-shaded areas in Figure 7c. During reaching, the neural activity observed earlier than the movement onset was consecutively highlighted over the movement period, as indicated by the green-shaded areas. The attention weights implicitly revealed thinner line segments than those in the LSTM-based neural decoder, suggesting that the GRU highlights fewer timesteps compared with the LSTM at each time bin for neural decoding. For monkey N, a few recently observed neural activities were highlighted during the preparatory and arm acceleration periods, as indicated by the green-shaded areas in Figure 7f. Compared with the LSTM, the GRU highlighted the neural activity observed at four recent timesteps rather than those at farther timesteps (i.e., the early preparatory period indicated by green arrows in Figure 7e). Similar to the vanilla RNN and LSTM, the GRU highlighted the most recent neural activity during the arm deceleration period (release phase).

## 5. Discussion

### 5.1. Timestep Selection for Varying Recording Conditions

For monkey Indy, the $T^{*}$ slightly differed, which suggests that the $T^{*}$ may be slightly affected by *T* for the three RNN-based neural decoders; however, these $T^{*}$ values varied in a small range, with $T^{*}\in \left[2,4\right]$ for the vanilla RNN, $T^{*}\in \left[5,7\right]$ for the LSTM and $T^{*}\in \left[3,6\right]$ for the GRU. Overall, the proposed TTS selected essential timesteps even when the input neural sequences covered long periods.

For monkey N, the TTS selected more than seven timesteps for the LSTM and GRU. This result is consistent with that of the monkey Indy, where the timesteps required for the vanilla RNN were fewer than those required for LSTM and GRU. The numbers of selected timesteps of the LSTM and GRU were strongly sensitive to *T*.

Neural recordings are nonstationary [4]. For instance, a slight movement of the electrodes or changes in the electrode impedance can alter neural recordings [1,35]. These changes affect the mapping between the neural activity and kinematic states, which in turn modify the required timesteps for neural decoding. Regardless of the nonstationary nature of the neural recordings, the proposed TTS can select the essential timesteps, imbuing the neural decoder with robustness against varying recording conditions.

### 5.2. Computational Efficiency and Comparable Decoding Performance

It has been known that a long neural sequence may introduce stochastic noise that decreases the decoding performance. Therefore, a neural decoder may benefit from the appropriate number of timesteps. The proposed TTS not only reduces the number of timesteps for neural decoding but also preserves the decoding performance, which remains comparable to that of a state-of-the-art method.

The proposed TTS could save the computation time of iBCI operation. The reduced computational burden for training is important for the clinical application of iBCIs. In fact, neural decoders typically require daily calibration for long-term use owing to variations in the recording conditions, such as micro-motion of electrodes or changes in an electrode’s impedance. The reduced calibration time provides a substantial benefit for the user of being able to operate the iBCI without long waiting periods. Moreover, a short calibration time reduces the power consumption, facilitating clinical application in portable devices.

### 5.3. Interpretation of Attention Weights in TTS

The attention weights of the TAM in TTS indicated whether the neural activity at one timestep is important to the neural decoding. For the vanilla RNN-based neural decoder, the patterns of the attention weights for monkey Indy and monkey N differ. Monkey Indy performed the reaching task in a horizontal plane, whereas monkey N performed the instructed delayed reach-to-grasp task. This suggests that various behavioral tasks might result in different patterns of the attention weights. The patterns of the attention weights suggested that the previous neural activity is essential for neural decoding during the preparatory period and execution of the movement, whereas the most recent neural activity is essential during the release phase. This finding is consistent with observations made in [5,36,37,38], where preparatory neural activity served as an initial condition for subsequent activity patterns. For the instructed delayed reach-to-grasp task, all findings from the three RNN-based neural decoders suggest that the preparatory neural activity is essential during the preparatory period and the execution of the movement, whereas the most recent neural activity is essential during the release phase.

### 5.4. Limitation of TTS

Figure 4 reveals that the number of timesteps selected by the TTS is sensitive to *T* for LSTM and GRU-based neural decoders. Assigning *T* for robust timesteps selection would be a challenging task. Furthermore, Figure 6 reveals that the vanilla RNN-based neural decoder with reduced timesteps achieved a lower decoding performance than that of state-of-the-art methods. Our TTS fails to select essential timesteps for the vanilla RNN-based neural decoder. How to select timesteps which are robust to *T* and are effective for all neural decoders is left for future work.

## 6. Conclusions

In this paper, we proposed TTS to select a few essential timesteps for RNN-based neural decoders while reducing the adverse effects of stochastic noise embedded in long neural sequences. The proposed TTS incorporates a TAM to estimate the saliency and relative importance of the input neural activity at each timestep. When an iBCI learns the functional mapping between the neural activity and kinematic states, the TAM is expected to learn to highlight the essential timesteps that contribute to neural decoding. The attention mechanism improves the interpretability of the information in the timesteps. Experimental results demonstrate that the proposed TTS selects a few essential timesteps from a long neural sequence to accurately predict the kinematic states. Using the proposed TTS, RNN-based neural decoders outperformed state-of-the-art methods on two nonhuman primate datasets. Furthermore, the TTS reduced the computation time for offline training and online prediction in the three RNN-based neural decoders. When visualizing the attention weights of the TAM in TTS, the preparatory neural activity observed before the movement onset is consecutively highlighted over the movement period, whereas the most recent neural activity is highlighted during the resting state. Experimental results also revealed that the number of essential timesteps varied over long recording days owing to changes in the recording conditions. Potential future work may incorporate an adaptive mechanism into the proposed TTS to handle changes in the recording conditions for the long-term use of iBCI.

## Figures and Tables

**Table 1:** Summary of existing iBCIs that use RNNs as neural decoders. The iBCIs were implemented for a nonhuman primate model. The time windows corresponding to the number of timesteps is given in parentheses in column #T. In [1], the number of hidden states and timesteps were optimized over a predefined range. (GRU, Gated recurrent unit; #L, Number of layers; #H, Number of hidden units; #T, Number of timesteps; DR: Dropout rate).

Study	Year	Decoded Kinematics	Neural Modality	Neural Decoder	#L	#H	#T	DR
Naufel et al. [13]	2018	Wrist electromyography	Spikes	LSTM	1	–	10 (500 ms)	0.25
Wang et al. [9]	2018	Hindlimb kinematics	Spikes	LSTM	–	200	3 (130 ms)	0.2
Tseng et al. [6]	2019	Hindlimb kinematics	Spikes	LSTM	2	128	30 (1500 ms)	0.2
Ahmadi et al. [16]	2019	Velocity	Spikes, local field potentials	LSTM	1	100	2 (256 ms)	0.2
Park et al. [17]	2019	Velocity	Spikes	LSTM	–	–	3 (150 ms)	–
Shaikh et al. [1]	2020	Actions	Spikes	LSTM	1	75–200	1–4 (600–900 ms)	0
Ahmadi et al. [8]	2021	Velocity	Spikes	Quasi-RNN	1	400	2 (256 ms)	0.4

**Table 2:** Settings of three RNN-based neural decoders widely used in existing iBCIs. All the decoders were implemented with layer normalization.

Parameter	RNN	LSTM	GRU
Number of hidden layers	2	2	2
Number of hidden states	128	256	128
Processing	Bidirectional		
Mini-batch size	32	256	64
Learning rate	1 × 10^−4^		
Number of weights	0.42 M	1.56 M	1.19 M

**Table 3:** Computation time of neural decoders using neural activity with T and $T^{*}$ timesteps. The efficiency is given by the computation time (ms) required to complete one epoch of offline training and one online prediction. The reduced number of timesteps $T^{*}$ of vanilla RNN, LSTM, and GRU were obtained from Figure 4, and the corresponding computation time was obtained over 37 sessions. The reduction in the computation time (in percentage) compared to that of T=10 is shown in parentheses. $D_{\mathrm{BO}}$ and $D_{\mathrm{TTS}}$ represent the time duration (second) of the timesteps selection achieved by the Bayesian optimization and our TTS, respectively. The reduction of the time duration (in percentage) compared to that of the Bayesian optimization is shown in parentheses.

	T=10		$T^{*}$		$D_{BO}$	$D_{TTS}$
	Training	Testing	Training	Testing		
Vanilla RNN	890.43 ± 5.21	1.08 ± 0.02	827.73 ± 6.76 (−7.04%)	0.88 ± 0.02 (−18.52%)	253.54 ± 32.24	45.66 ± 3.05 (−81.99%)
LSTM	219.87 ± 51.22	1.51 ± 0.08	191.85 ± 44.08 (−12.74%)	1.26 ± 0.07 (−16.56%)	244.88 ± 12.43	81.41 ± 2.74 (−66.76%)
GRU	1006.02 ± 6.18	1.20 ± 0.02	948.67 ± 13.77 (−5.70%)	1.00 ± 0.03 (−16.67%)	211.67 ± 7.38	108.61 ± 2.76 (−48.69%)

## Data Availability

Publicly available datasets were analyzed in this study. This data can be found here: https://zenodo.org/record/583331 (accessed on 1 September 2020) for monkey Indy and https://gin.g-node.org/INT/multielectrode_grasp (accessed on 1 September 2020) for monkey N.
